# Modelling impact and cost‐effectiveness of oral pre‐exposure prophylaxis in 13 low‐resource countries

**DOI:** 10.1002/jia2.25451

**Published:** 2020-02-28

**Authors:** Carel Pretorius, Melissa Schnure, Juan Dent, Robert Glaubius, Guy Mahiane, Matthew Hamilton, Meghan Reidy, Sindy Matse, Emmanuel Njeuhmeli, Delivette Castor, Katharine Kripke

**Affiliations:** ^1^ Avenir Health Glastonbury CT USA; ^2^ The Palladium Group Washington DC USA; ^3^ Avenir Health Takoma Park MD USA; ^4^ Ministry of Health Mbabane Eswatini; ^5^ United States Agency for International Development (USAID) Mbabane Eswatini; ^6^ Office of HIV/AIDS USAID Washington DC USA

**Keywords:** PrEP, pre‐exposure prophylaxis, cost‐effectiveness, mathematical modelling, HIV, policy

## Abstract

**Introduction:**

Oral pre‐exposure prophylaxis (PrEP) provision is a priority intervention for high HIV prevalence settings and populations at substantial risk of HIV acquisition. This mathematical modelling analysis estimated the impact, cost and cost‐effectiveness of scaling up oral PrEP in 13 countries.

**Methods:**

We projected the impact and cost‐effectiveness of oral PrEP between 2018 and 2030 using a combination of the Incidence Patterns Model and the Goals model. We created four PrEP rollout scenarios involving three priority populations—female sex workers (FSWs), serodiscordant couples (SDCs) and adolescent girls and young women (AGYW)—both with and without geographic prioritization. We applied the model to 13 countries (Eswatini, Ethiopia, Haiti, Kenya, Lesotho, Mozambique, Namibia, Nigeria, Tanzania, Uganda, Zambia and Zimbabwe). The base case assumed achievement of the Joint United Nations Programme on HIV/AIDS 90‐90‐90 antiretroviral therapy targets, 90% male circumcision coverage by 2020 and 90% efficacy and adherence levels for oral PrEP.

**Results:**

In the scenarios we examined, oral PrEP averted 3% to 8% of HIV infections across the 13 countries between 2018 and 2030. For all but three countries, more than 50% of the HIV infections averted by oral PrEP in the scenarios we examined could be obtained by rollout to FSWs and SDCs alone. For several countries, expanding oral PrEP to include medium‐risk AGYW in all regions greatly increased the impact. The efficiency and impact benefits of geographic prioritization of rollout to AGYW varied across countries. Variations in cost‐effectiveness across countries reflected differences in HIV incidence and expected variations in unit cost. For most countries, rolling out oral PrEP to FSWs, SDCs and geographically prioritized AGYW was not projected to have a substantial impact on the supply chain for antiretroviral drugs.

**Conclusions:**

These modelling results can inform prioritization, target‐setting and other decisions related to oral PrEP scale‐up within combination prevention programmes. We caution against extensive use given limitations in cost data and implementation approaches. This analysis highlights some of the immediate challenges facing countries—for example, trade‐offs between overall impact and cost‐effectiveness—and emphasizes the need to improve data availability and risk assessment tools to help countries make informed decisions.

## 1 Introduction

A growing body of evidence has shown oral pre‐exposure prophylaxis (PrEP)—the use of oral antiretroviral drugs (ARVs) by HIV‐negative individuals to prevent HIV acquisition—to be safe and effective in reducing risk of HIV infection in several populations 1, 2, 3, 4, 5, 6. As of 2015, World Health Organization (WHO) guidelines recommend offering PrEP to all those at “substantial risk” of HIV infection, roughly defined as populations with HIV incidence at or greater than 3% 7. The U.S. President's Plan for Emergency AIDS Relief (PEPFAR) has identified PrEP as a priority intervention for individuals at substantial risk, including adolescent girls and young women (AGYW) in high HIV prevalence settings, serodiscordant couples (SDCs), female sex workers (FSWs), men who have sex with men (MSM) and people who inject drugs (PWID) 8.

Identifying priority populations and individuals “at substantial risk” of HIV infection for PrEP provision, and target setting and budgeting for oral PrEP scale‐up, remain almost universal challenges. This is due, in no small part, to the difficulty of measuring or estimating HIV incidence at subnational levels 9. WHO suggests that risk scores can potentially be used to assess individuals in need of PrEP. However, these scores have not performed consistently across multiple settings 10. We developed a new mathematical modelling approach and used it to estimate the impact, cost and cost‐effectiveness of oral PrEP scale‐up scenarios in 13 countries at different stages of PrEP delivery: Eswatini, Ethiopia, Haiti, Kenya, Lesotho, Malawi, Mozambique, Namibia, Nigeria, Tanzania, Uganda, Zambia and Zimbabwe. These countries in sub‐Saharan Africa and the Caribbean were selected as they have relatively high HIV burden, were at policy or programme development stages of rolling out PrEP, had not had extensive modelling for PrEP, and were prioritized by PEPFAR as long‐term strategy countries for achieving epidemic control 11 through UNAIDS Fast‐Track goals 12. These countries represent 39% of the global burden of HIV 13. PEPFAR is the largest funding initiative against a single disease in modern history and is focused on HIV implementation in low‐resource settings. This analysis can be used to inform prioritization, target setting, and other decisions related to oral PrEP scale‐up.

## 2 Methods

### 2.1 Country consultations

To better understand the key PrEP policy and implementation questions that can be addressed with mathematical modelling, the modelling team (CP, MS, JD, MH, MR, DC, KK) held in‐depth consultations in four countries at different stages of oral PrEP rollout (Eswatini, Lesotho, Mozambique and Uganda). These countries were chosen because key country stakeholders expressed interest in using mathematical modelling to inform their strategic plans for rolling out oral PrEP. PrEP was expected to be introduced within the context of delivering combination HIV prevention interventions and achieving Fast‐Track goals. The priority populations most commonly considered by the countries for PrEP rollout were AGYW aged 15 to 24 years in high‐incidence settings, FSWs and SDCs.

### 2.2 Modelling approach

Country consultations produced two thematic modelling questions that we addressed in this analysis: (1) how different scenarios for rolling out oral PrEP by subpopulation and geography affect the impact and cost‐effectiveness, and (2) how impact and cost‐effectiveness of oral PrEP change under different assumptions of scale‐up of antiretroviral therapy (ART) and voluntary medical male circumcision (VMMC). This paper applies these questions to the 13 countries mentioned above.

**Figure 1:**
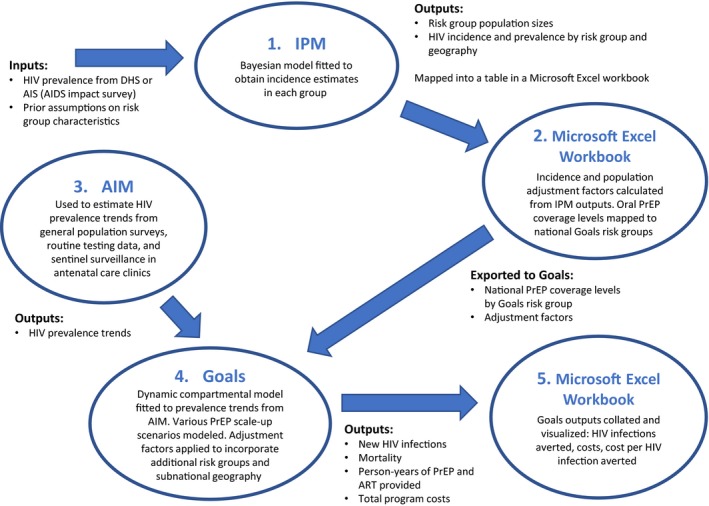
Process for linking AIM, Goals and IPM used in the modelling approach. The figure shows the inputs, outputs and steps used to link the three modelling tools in this modelling exercise to generate the results presented in this article.

We adapted and integrated three existing modelling tools (Figure 1) to estimate the impact, cost and cost‐effectiveness: the AIDS Impact Model (AIM) and the Goals model from the open source Spectrum suite of policy tools 14, 15, 16 and the Incidence Patterns Model (IPM) 17. 2016 UNAIDS AIM files 18 were used to estimate HIV prevalence trends from general population surveys, routine testing data 15, 19 and sentinel surveillance in antenatal care clinics. These files were validated by each country's health ministry. We used these HIV prevalence trends to calibrate the Goals model, a dynamic, compartmental model, and used Goals to estimate the impact of HIV prevention and treatment interventions. Goals' risk group structure includes low‐ and medium‐risk heterosexuals, FSWs and their clients, MSM and PWID. It does not include AGYW or SDCs, nor does it easily lend itself to subnational analyses. In contrast, IPM can produce estimates of HIV infections by subpopulation, age band and geography. We therefore used IPM to produce incidence and population size adjustment factors that allowed us to disaggregate the Goals risk groups to include SDCs, AGYW and subnational geography in the Goals analyses (see supporting information File S1 for details). Both IPM and Goals produced compatible overall incidence estimates in the 2017 base year (for Goals modelled incidence in 2017 see supporting information File S2).

**Figure 2:**
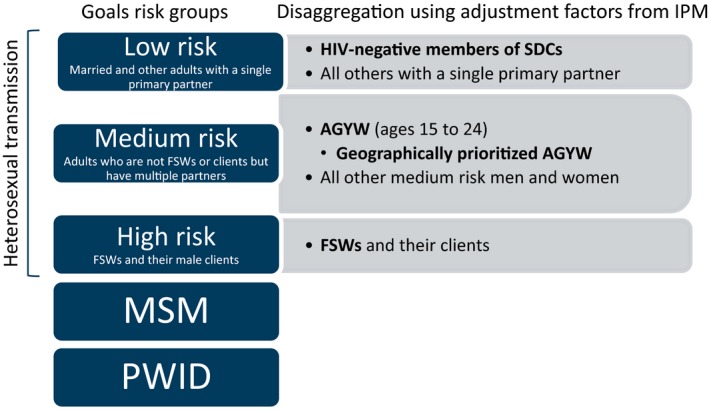
Disaggregation of Goals risk groups. The figure shows the definitions of non‐overlapping risk groups used in the modelling exercise. Populations included in this modelling analysis are shown in bold.

The use of a compartmental model requires the definition of non‐overlapping risk groups (Figure 2). SDCs were incorporated as a subset of the “low‐risk” group in Goals, because in the model this group represents people with one primary heterosexual partner. SDCs are not actually low risk; the IPM adjustment factors are used to adjust the expected HIV infections averted based on how much higher the risk of SDCs is than the rest of the population who are in stable heterosexual partnerships. For SDCs, PrEP use was assumed to last for six months in the HIV‐negative partner, concurrent with ART initiation and achieving viral suppression in the HIV‐positive partner.

AGYW, defined as women aged 15 to 24 years, were incorporated into the Goals “medium‐risk” group, which represents heterosexual adults with multiple partners; in the model this group does not include FSWs or women in SDCs. While in reality AGYW will actually span the low‐, medium‐ and high‐risk groups, we incorporated them into the medium‐risk group in the model to indicate that those AGYW provided with PrEP would be selected in some way to be at higher risk than the general population. It was assumed that AGYW who were sex workers would be reached through the FSW programmes and AGYW in serodiscordant partnerships would be reached through SDC programmes, so these groups were not treated as separate populations in the model. Medium‐risk AGYW were further disaggregated in this modelling exercise to allow for geographic prioritization.

MSM and PWID were not included in this modelling exercise, as available data were insufficient to produce reliable estimates of population size and HIV incidence for all 13 countries.

The average number of partners and the size of the Goals medium‐risk group were based on Demographic and Health Survey (DHS) data for each country regarding numbers of partners and sex acts. Given the subjectivity of survey responses regarding sexual behaviour, these parameters were subsequently adjusted as needed to calibrate each model to HIV prevalence trends. See Supporting Information File S1 for details on the modelling approach.

### 2.3 Cost analysis and unit cost data

As published primary oral PrEP unit cost data were unavailable for most countries, unit costs (provider perspective) were adapted from a facility‐based costing study conducted in Kenya at three sites serving FSWs and MSM 20. Table 1 shows extrapolated oral PrEP unit costs per person per year (based on the Kenya study) used in our analysis. Cost categories included training, adherence, demand generation, laboratory, personnel, ARVs (e.g. emtricitabine/tenofovir), equipment and facility costs. Above site‐level costs were not included (see Supporting Information File S3 for additional explanation of unit cost derivation).

**Table 1:** Unit costs^a^

Country	Oral PrEP Unit Cost	ART Unit Cost, Adults
Ethiopia	106	190
Haiti	117	625
Malawi	121	250
Zimbabwe	121	254
Mozambique	133	260
Uganda	133	467
Lesotho	148	297
Tanzania	164	480
Zambia	170	292
Eswatini	195	513
Kenya	206	257
Nigeria	221	819
Namibia	236	673

Sources: ART costs 16; PrEP costs described in text.

a Costs are shown in 2017 USD.

### 2.4 Oral PrEP rollout scenarios

Based on country consultations and available data, the team created four scenarios to illustrate the potential benefits and trade‐offs of providing PrEP to progressively broader populations (Table 2).

**Table 2:** Oral PrEP rollout scenarios

Scenario	Description
Scenario 1	National rollout for FSWs
Scenario 2	National rollout for FSWs + SDCs
Scenario 3	National rollout for FSWs + SDCs + geographically prioritized medium‐risk AGYW
Scenario 4	National rollout for FSWs + SDCs + medium‐risk AGYW in all provinces/regions (no geographic prioritization)

In Scenario 3, oral PrEP was assumed to be provided to AGYW in all subnational regions/provinces with higher than median HIV incidence among AGYW. PrEP provision for FSWs and SDCs was not geographically prioritized, based on an assumption of high incidence regardless of location. All four scenarios assumed an s‐shaped scale‐up pattern starting at 0% coverage in 2017 and reaching 50% coverage of each indicated population in 2030 (illustrated in Supporting Information File S4). For consistency across all 13 countries and to be consistent with the optimistic assumptions used for ART and VMMC uptake and effectiveness, we assumed optimistic levels (90%) for both the efficacy of and adherence to oral PrEP for all four scenarios (Table 3).

**Table 3:** Parameters and sources

Parameter	Value	Data source	Notes
PrEP efficacy	90%	Assumption, Partners Demonstration Project 21	Optimistic, intended to be consistent with optimistic ART and VMMC assumptions
PrEP adherence	90%	Assumption, 22, 23	Optimistic, intended to be consistent with optimistic ART and VMMC assumptions
PrEP coverage	S‐shaped curve starting at 0% coverage in 2017 and plateauing at 50% coverage by 2030	Assumption	See Supporting Information File S4 for an illustration of the coverage levels over time.
Reduction of infectiousness with ART to without ART	0.25 from beginning of model until 2010; linear interpolation from 0.25 in 2010 to 0.1 in 2020; linear interpolation from 0.1 in 2020 to 0.05 in 2030	Assumption, based on the achievements of the UNAIDS Fast‐Track targets	This parameter represents the effect of viral suppression on transmission of HIV; a value of 0.25 indicates that the population is 75% virally suppressed.
ART scale‐up, base (90‐90‐90 by 2020, Fast‐Track) scenario	Linear interpolation from 2017 levels (See Table S1.2 in Supporting Information File S1) to 81% coverage in 2020, followed by linear interpolation from 81% coverage in 2020 to 90% coverage in 2030.	Coverage values through 2017 are estimated in AIM based on programme service statistics provided and validated as part of the annual AIM file update process. After 2017, assumption, based on the achievements of the UNAIDS Fast‐Track targets	This parameter combines the 90% testing and 90% on treatment targets. 90% x 90% = 81%. The targets for 2030 are 95% and 95%; 95% x 95% = 90%.
ART scale‐up, constant coverage scenario	Keep ART coverage constant at 2017 levels	Assumption	
ART scale‐up, 90‐90‐90 by 2030 scenario	Linear interpolation from 2017 levels (See Table S1.2 in Supporting Information File S1) to 81% coverage in 2030.	Assumption, based on delayed achievement of the Fast‐Track targets	This parameter combines the 90% testing and 90% on treatment targets. 90% x 90% = 81%.
VMMC efficacy	60%	24, 25, 26	Reduction in male susceptibility to HIV infection when circumcised
VMMC scale‐up, base (90 by 2020, Fast‐Track) scenario	Linear interpolation from 2017 levels (See Table S1.2 in Supporting Information File S1) to 90% coverage by 2020; 90% coverage after 2030.	Prior to 2017, values are extracted from DHS and AIS through http://statcompiler.com. After 2017, assumption based on UNAIDS VMMC targets.	
VMMC scale‐up, constant coverage scenario	Keep male circumcision (MC) coverage constant at 2017 levels	Assumption	
VMMC scale‐up, 90 by 2030 scenario	Linear interpolation from 2017 levels (See Table S1.2 in Supporting Information File S1) to 90% coverage by 2030	Assumption, based on delayed achievement of the Fast‐Track targets	

### 2.5 Impact: HIV infections averted

Impact was reported as the cumulative number of HIV infections averted between 2018 and 2030, inclusive. To estimate this, we compared the number of HIV infections in the rollout scenarios to a counterfactual scenario with the same ART and VMMC scale‐up, but without oral PrEP scale‐up. Unless otherwise stated, analyses were based on a counterfactual scenario in which UNAIDS 90‐90‐90 ART and VMMC targets are achieved by 2020 (Table 3). Percent impact was defined as the number of HIV infections averted by rolling out oral PrEP divided by the total number of HIV infections without PrEP (Formula 1):

Formula 1. Percent impact from oral PrEP

$$\frac{\text{New}\;\text{infections}\;\text{without}\;\text{oral}\;\text{PrEP}-\text{New}\;\;\text{infections}\;\text{with}\;\text{oral}\;\text{PrEP}}{\text{New}\;\text{infections}\;\text{without}\;\text{oral}\;\text{PrEP}}$$

### 2.6 Cost‐effectiveness

Cost‐effectiveness was reported as the incremental cost per HIV infection averted between 2018 and 2030. The incremental cost of oral PrEP was estimated by comparing HIV programme costs in oral PrEP rollout scenarios (including the cost of all HIV prevention and treatment interventions 16) versus counterfactual scenarios. Incremental cost included cost savings from ART not needed due to HIV infections averted from oral PrEP (2018 to 2030). The cost per HIV infection averted was calculated by dividing the number of infections averted by the incremental cost of oral PrEP (Formula 2):

Formula 2: Cost per HIV infection averted

$$\frac{\text{Cost}\;\text{of}\;\text{HIV}\;\text{programme}\;\text{including}\;\text{oral}\;\text{PrEP}-\text{Cost}\;\text{of}\;\text{HIV}\;\text{programme}\;\text{without}\;\text{oral}\;\text{PrEP}}{\text{New}\;\;\text{infections}\;\text{without}\;\text{oral}\;\text{PrEP}-\text{New}\;\text{infections}\;\;\text{with}\;\;\text{oral}\;\;\text{PrEP}}$$

### 2.7 Ethical considerations

As patient records were not collected or reviewed, this study did not require institutional review board clearance. Only publicly available and previously published data were used in this modelling exercise.

## 3 Results

### 3.1 Overall impact of oral PrEP

The total impact of Scenario 4—rollout of PrEP to FSWs, SDCs and AGYW in all provinces/regions—across the 13 countries in the context of achieving the Fast‐Track targets for ART and VMMC was approximately 230,000 (6%) HIV infections averted (2018 to 2030). Sensitivity analyses keeping ART and VMMC coverage at 2017 levels (data shown in Supporting Information File S5) estimated impact to exceed 700,000 HIV infections averted across the 13 countries over the same period.

**Figure 3:**
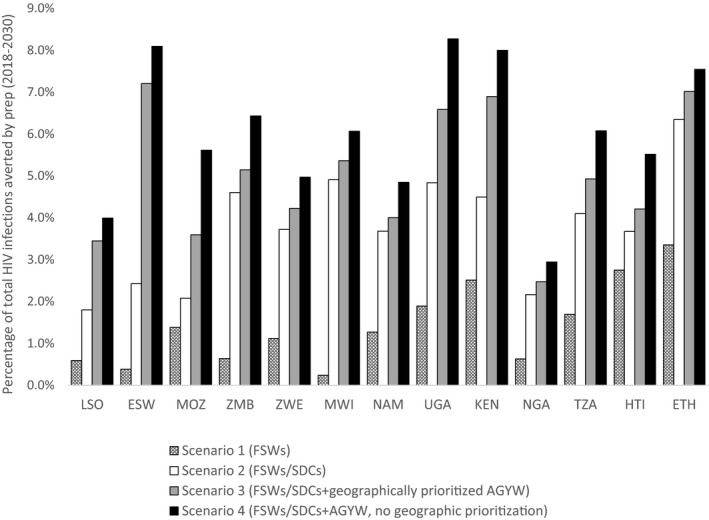
Percentage of HIV infections averted by oral PrEP under different rollout scenarios. The graph shows the relative impact (percentage of HIV infections averted) of different oral PrEP rollout scenarios in the context of UNAIDS Fast‐Track achievement. Countries are listed from left to right in decreasing order of HIV incidence in 2017 among women aged 15 to 49 years (source: Goals files). AGYW, adolescent girls and young women; KEN, Kenya; ESW, Eswatini; ETH, Ethiopia; FSWs, female sex workers; HTI, Haiti; LSO, Lesotho; MWI, Malawi; MOZ, Mozambique; NAM, Namibia; NGA, Nigeria; PrEP, pre‐exposure prophylaxis; SDCs, serodiscordant couples; TZA, Tanzania; UGA, Uganda; ZMB, Zambia; ZWE: Zimbabwe.

**Figure 4:**
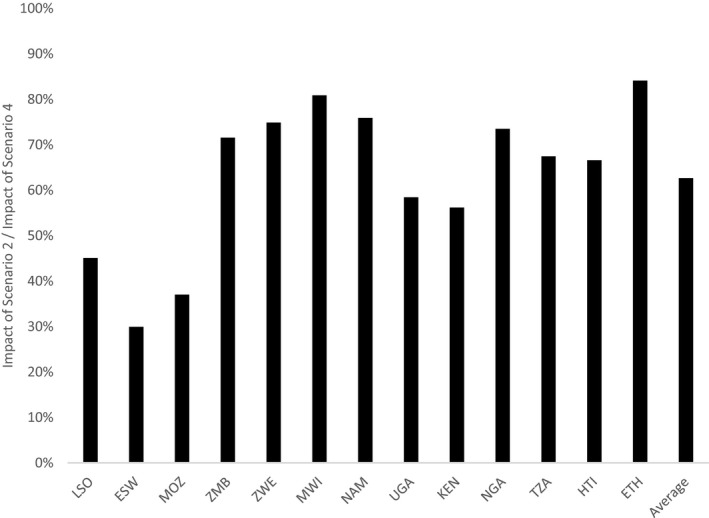
Impact of focused oral PrEP rollout to FSWs and SDCs relative to expanded rollout to FSWs, SDCs and AGYW (nationally). The graph shows the impact (HIV infections averted) of focused rollout to FSWs and SDCs (Scenario 2) divided by the impact of an expanded rollout to FSWs, SDCs and medium‐risk AGYW in all regions (Scenario 4). ESW, Eswatini; ETH, Ethiopia; HTI, Haiti; KEN, Kenya; LSO, Lesotho; MOZ, Mozambique; MWI, Malawi; NAM, Namibia; NGA, Nigeria; TZA, Tanzania; UGA, Uganda; ZMB, Zambia; ZWE, Zimbabwe.

**Figure 5:**
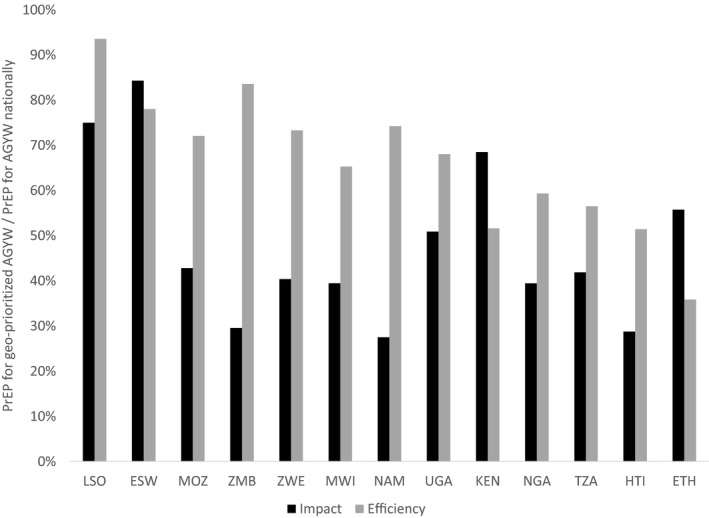
Impact and efficiency of geographically prioritized rollout of oral PrEP for AGYW relative to national rollout for AGYW. The graph shows the impact (HIV infections averted) and efficiency (person‐years of oral PrEP per HIV infection averted) of geographically prioritized rollout of oral PrEP to medium‐risk AGYW in regions with higher than median HIV incidence divided by those same metrics for oral PrEP rollout to medium‐risk AGYW in all regions. AGYW, adolescent girls and young women; ESW, Eswatini; ETH, Ethiopia; HTI, Haiti; KEN, Kenya; LSO, Lesotho; MOZ, Mozambique; MWI, Malawi; NAM, Namibia; NGA, Nigeria; PrEP, pre‐exposure prophylaxis; TZA, Tanzania; UGA, Uganda; ZMB, Zambia; ZWE, Zimbabwe.

Figure 3 shows the percent impact of the four oral PrEP rollout scenarios. For countries such as Lesotho, Eswatini, Zambia and Malawi with highly generalized epidemics, providing oral PrEP only to FSWs (Scenario 1) had relatively low impact, despite high HIV risk among this population (for estimated HIV incidence in each country in 2017, see Supporting Information File S2). In most countries, providing oral PrEP to SDCs provided substantial additional benefit. The additional benefits of including AGYW, with or without geographic prioritization, are considered in detail in Figures 4 and 5.

### 3.2 What proportion of all HIV infections averted by oral PrEP can be averted by providing oral PrEP to FSWs and SDCs?

Figure 4 shows the relative impact of Scenario 2 (focused rollout of oral PrEP to FSWs and SDCs) compared with Scenario 4 (expanded rollout to FSWs, SDCs and AGYW without geographic prioritization), expressed as the number of HIV infections averted in Scenario 2 divided by the number of infections averted in Scenario 4. In 8 of the 13 countries (Zambia, Zimbabwe, Malawi, Namibia, Nigeria, Tanzania, Haiti and Ethiopia), Scenario 2 achieved more than 60% of the impact of Scenario 4.

### 3.3 How much does geographic prioritization contribute to the total impact and efficiency of providing oral PrEP to AGYW?

Figure 5 shows the impact and efficiency—person‐years of oral PrEP per HIV infection averted—of oral PrEP for geographically prioritized medium‐risk AGYW as proportions of those same metrics for oral PrEP provided to AGYW nationally. This analysis shows that the geographic prioritization could achieve more than 50% of the total impact in countries like Lesotho, Eswatini, Kenya and Ethiopia. For example, in Lesotho and Eswatini, providing oral PrEP to AGYW in regions with higher than median HIV incidence was projected to avert 75% and 84% respectively of HIV infections projected to be averted by providing oral PrEP to AGYW in all regions. However, for these two countries, efficiency gains from geographic prioritization were minimal, suggesting that while HIV incidence among AGYW in these countries is relatively consistent across regions, the population is more densely concentrated in regions with higher than median HIV incidence.

Countries with more concentrated or geographically focused epidemics—Kenya, Nigeria, Tanzania, Haiti and Ethiopia—achieved more pronounced efficiency gains (lower person‐years of oral PrEP per HIV infection averted) by geographic prioritization of oral PrEP for AGYW.

### 3.4 How do the efficiency and cost‐effectiveness of oral PrEP rollout for FSWs and SDCs vary by country?

**Figure 6:**
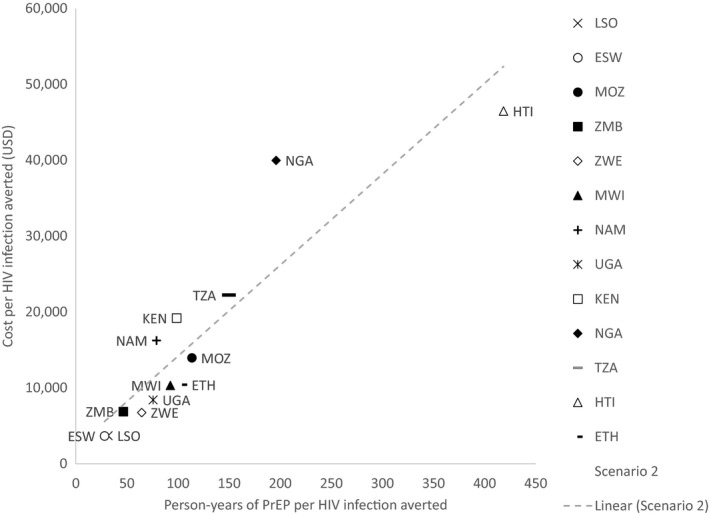
Efficiency (person‐years of oral PrEP per HIV infection averted) and cost‐effectiveness (cost per HIV infection averted) of oral PrEP rollout to FSWs and SDCs (Scenario 2). The dashed line represents a linear trendline across all points. The legend shows the countries ranked in order of HIV incidence among women aged 15 to 49 years from highest incidence at the top to lowest at the bottom. ESW, Eswatini; ETH, Ethiopia; HTI, Haiti; KEN, Kenya; LSO, Lesotho; MOZ, Mozambique; MWI, Malawi; NAM, Namibia; NGA, Nigeria; PrEP, pre‐exposure prophylaxis; TZA, Tanzania; UGA, Uganda; ZMB, Zambia; ZWE, Zimbabwe.

As the cost of oral PrEP is expected to vary by country, we examined the efficiency (person‐years of oral PrEP per HIV infection averted) of oral PrEP separately from cost‐effectiveness (cost per HIV infection averted). Figure 6 shows how efficiency and cost‐effectiveness for Scenario 2 (FSWs and SDCs) vary by country, with fewer person‐years of oral PrEP per HIV infection averted representing greater efficiency and lower cost per HIV infection averted representing greater cost‐effectiveness. With some notable exceptions, such as Mozambique and Ethiopia, efficiency rankings roughly corresponded with overall HIV incidence rankings among women aged 15 to 49 years—indicated by the order in which the countries appear in the figure legend. Unsurprisingly, countries with the highest HIV incidence, such as Lesotho and Eswatini, had the greatest efficiency and cost‐effectiveness for rolling out oral PrEP to FSWs and SDCs. In Nigeria, Kenya and Namibia, which have among the highest estimated oral PrEP unit costs, oral PrEP rollout to these populations cost more per HIV infection averted than was indicated by efficiency alone. Conversely, in Haiti, which has one of the lowest unit costs, oral PrEP rollout was more cost‐effective than efficiency suggested.

### 3.5 Oral PrEP rollout in the context of combination prevention

**Figure 7:**
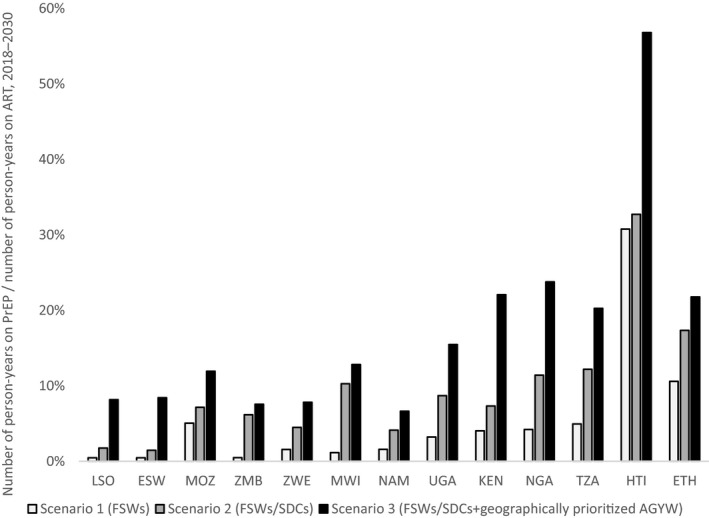
Person‐years of oral PrEP relative to person‐years on ART. Shows the number of person‐years on PrEP for each scenario/country divided by the number of person‐years on ART for that country, 2018 to 2030. AGYW, adolescent girls and young women; ART, antiretroviral therapy; ESW, Eswatini; ETH, Ethiopia; HTI, Haiti; KEN, Kenya; LSO, Lesotho; MOZ, Mozambique; MWI, Malawi; NAM, Namibia; NGA, Nigeria; PrEP, pre‐exposure prophylaxis; SDC, serodiscordant couple; TZA, Tanzania; UGA, Uganda; ZMB, Zambia; ZWE, Zimbabwe.

Figure 7 shows the number of person‐years of oral PrEP required to reach coverage targets relative to the number of person‐years of ART, 2018 to 2030. Understanding this relationship is useful for high‐level supply chain and implementation planning, as PrEP and ART programmes may involve an integrated service delivery and/or shared ART regimens. For example, in Lesotho the number of person‐years of PrEP for Scenario 3 was less than 10% of the number of person‐years of ART. Moving from left to right in the figure, oral PrEP was projected to represent an increasing proportion of the overall ARV supply in less generalized HIV epidemics. For most countries, rolling out oral PrEP to FSWs, SDCs and geographically prioritized AGYW (Scenario 3) was not projected to have a substantial impact on the supply chain for ARVs. However, in countries with more concentrated or geographically focused epidemics, such as Kenya, Nigeria and Haiti, adding AGYW, even geographically prioritized medium‐risk AGYW, required a substantial increase in the ARV supply.

### 3.6 Sensitivity analyses: scale‐up of ART and VMMC

**Figure 8:**
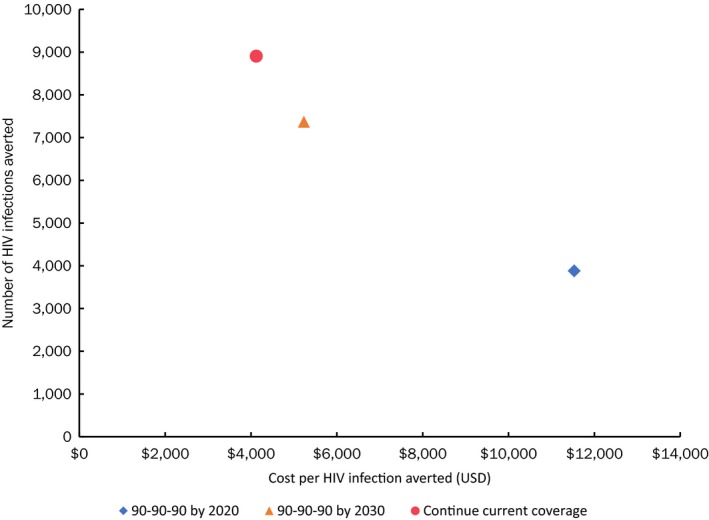
Number of HIV infections averted and cost per infection averted due to oral PrEP scale‐up (Scenario 4, rollout to FSWs, SDCs and medium‐risk AGYW in all regions) from 2018 to 2030 by treatment scenario.

While most of these countries have adopted the Fast‐Track targets as part of their national HIV strategies, few of them are likely to achieve these targets for both ART and VMMC by 2020, based on the latest UNAIDS country data 13. We conducted a sensitivity analysis to assess how different scenarios for scaling up ART and VMMC would change the impact and cost‐effectiveness of rolling out oral PrEP to the populations in rollout scenario 4 (FSWs, SDCs and medium‐risk AGYW in all regions). The first two scenarios assumed 90% male circumcision (MC) coverage among males ages 10 to 29 years and achievement of the 90‐90‐90 ART targets by either 2020 or 2030. A third scenario assumes MC and ART coverage stay at 2017 levels. Illustrative results for Eswatini are shown in Figure 8. This figure demonstrates that oral PrEP is more cost‐effective and averts more HIV infections if ART and VMMC scale‐up efforts do not reach their targets as planned. The “continue current (ART and VMMC) coverage” scenario provides a lower bound on the cost per HIV infection averted by oral PrEP, although it is important to remember that oral PrEP will likely be integrated into ART programmes, so oral PrEP scale‐up is unlikely to outpace ART scale‐up. Achieving the ART and VMMC targets by 2030 instead of 2020 more than halves the cost per HIV infection averted from US$12,000 to US$5,000. Moreover the delay nearly doubles the number of infections averted from oral PrEP scale‐up from 3,900 to 7,400. Similar trends were seen in the other 12 countries (see Supporting Information File S5).

### 3.7 Sensitivity analyses: cost by priority population

There have been few facility‐based studies of the cost of delivering oral PrEP published to date, and none that assess the potential differences in cost by risk group. We estimated the effect of varying oral PrEP unit costs by risk group, representing potential differences in costs such as for demand generation and adherence for different risk groups, on the ranking of risk groups by cost‐effectiveness. Tables 4 and 5 show illustrative results for Eswatini of cost‐effectiveness by risk group when the oral PrEP unit cost is US$195 and US$293 (1.5‐fold higher). The order of cost‐effectiveness by risk group does not change even if either FSW or the other populations have higher unit costs, demonstrating that the cost‐effectiveness ranking is robust to this level of variation in unit cost, as seen in Tables 4 and 5. Similar trends were seen in the other 12 countries (not shown).

**Table 4:** Sensitivity analysis of higher cost for reaching FSWs

Risk group	Oral PrEP unit cost	Cost per HIV infection averted
SDCs	$195	$2,655
FSWs	$293	$13,674
AGYW	$195	$14,535

Costs are shown in 2017 USD.

**Table 5:** Sensitivity analysis of higher cost for reaching general population groups

Risk group	Oral PrEP Unit Cost	Cost per HIV infection averted
SDCs	$293	$3,983
FSWs	$195	$9,116
AGYW	$293	$21,803

Costs are shown in 2017 USD.

## 4 Discussion

Sub‐Saharan African and Caribbean countries are beginning to introduce oral PrEP into their HIV prevention portfolios in a context of rapid scale‐up of ART and limited or stagnant HIV budgets. As such, they are looking for guidance as to how to best prioritize which populations should receive PrEP and how this guidance applies to their specific country epidemic context. A key question of interest is whether PrEP should be provided to AGYW, whose HIV incidence is high, but not as high as the incidence among other high‐risk populations, such as FSWs and SDCs. In response to these questions, we assessed the impact (number and percent of HIV infections averted) and cost‐effectiveness of four rollout scenarios for oral PrEP in 13 sub‐Saharan African and Caribbean countries, as well as the impact of geographic prioritization of AGYW. For most of the countries included in this analysis, our results suggest that providing oral PrEP only to FSWs has relatively low impact and that substantial additional benefits could be gained by expanding rollout to include SDCs. For all but Eswatini, Lesotho and Mozambique, our analysis suggests that more than 50% of HIV infections averted by the oral PrEP scenarios we examined were obtained by providing oral PrEP to FSWs and SDCs. For those three countries and, to a lesser extent, for Kenya and Uganda, expanding oral PrEP rollout to include medium‐risk AGYW in all regions greatly increased the impact. Overall, the benefits of geographic prioritization of AGYW varied across countries in terms of both efficiency and impact. Not surprisingly, countries with more concentrated or geographically focused epidemics achieved more pronounced efficiency gains by geographic prioritization of oral PrEP for AGYW. The efficiency and cost‐effectiveness of Scenario 2—providing oral PrEP to FSWs and SDCs—varied across countries, roughly aligning with HIV incidence among adult women. The alignment of cost‐effectiveness with HIV incidence can be somewhat modified by country‐specific variations in the costs of oral PrEP provision. During our country consultations, countries expressed concern that oral PrEP rollout could have substantial impacts on the ARV supply chain. However, for most of the countries, for the scenarios we examined, this concern was not borne out by the results of this modelling exercise. In the scenarios presented here, the cost‐effectiveness of oral PrEP was shown to increase when ART and VMMC scale‐up was delayed. While previous modelling has shown that oral PrEP is projected to be more cost‐effective when scaled up prior to ART 27, this scenario is unrealistic, since oral PrEP and ART require much of the same service delivery infrastructure.

This set of analyses builds on previous oral PrEP modelling studies. Several studies have demonstrated that oral PrEP is only cost‐effective when provided to individuals at very high risk of HIV infection 28. Others have ranked PrEP for cost‐effectiveness along with other HIV interventions within countries 29, 30. Verguet, et al^31^ examined the impact and cost‐effectiveness of PrEP for the general population across 42 sub‐Saharan African countries, finding that it was only cost‐effective for this population in countries with high HIV prevalence and low male circumcision prevalence. McGillen, et al 32 demonstrated that in the context of a fixed overall funding envelope, PrEP could avert the greatest number of HIV infections when prioritized by both individual risk and subnational geography. Our study focuses on 13 countries prioritized by PEPFAR for PrEP provision and examines the differences across them with respect to which populations should be prioritized for PrEP and how much subnational geographic incidence variation affects the impact and cost‐effectiveness of PrEP for AGYW. To the best of our knowledge, this is the first study that compares PrEP for SDCs and AGYW across countries. In addition, we examine trade‐offs between impact and cost‐effectiveness, where in a few countries, providing PrEP to the most cost‐effective populations results in relatively low impact.

### 4.1 Policy implications

#### 4.1.1 Cost‐effectiveness and efficiency

Cost‐effectiveness is affected by various factors, including the relative efficiency of different rollout scenarios and estimated unit cost of oral PrEP. For the countries included in our analysis, except Mozambique, the efficiency of Scenario 2 was roughly aligned with HIV incidence in the general adult population. However, for Eswatini and Lesotho, the efficiency of Scenario 2 stands in contrast to its relatively low impact compared with Scenarios 3 and 4.

Variations in cost‐effectiveness across countries reflected both differences in HIV incidence and expected variations in unit cost. Our results show that, in some settings, oral PrEP may be less cost‐effective than suggested by incidence and risk, while in other settings, the opposite is true. For instance, Namibia has relatively high HIV incidence but may have higher than average unit cost, resulting in worse than expected cost‐effectiveness. Whereas, in Ethiopia, where HIV incidence is below average, but the unit cost is also low, cost‐effectiveness is better than expected based on incidence alone. Cost‐effectiveness estimates will be improved as primary cost data are collected in more countries and studies are conducted to illuminate how costs vary by such factors as implementation model, setting and population served.

#### 4.1.2 Health system implications

Our analysis examining person‐years of oral PrEP relative to person‐years on ART provides insight into the health system implications of oral PrEP scale‐up, a major concern for stakeholders. Results show that, for most countries, providing PrEP to FSWs and SDCs (Scenario 2) would have minimal impact on the ARV supply chain. For countries with more concentrated or geographically focused epidemics, reaching an increasing proportion of AGYW, even when geographically or otherwise prioritized, will lead to oral PrEP occupying a larger portion of ARV commodities in the supply chain, requiring careful planning to ensure the necessary resources are available for both prevention and treatment. Rolling out oral PrEP will require a significant degree of planning to ensure the uninterrupted availability of ARVs for prevention and treatment.

### 4.2 Limitations

Our model did not include several factors that affect variation in risk, which likely affected the robustness of the results presented. Our method does not, for example, account for inter‐regional sexual mixing (e.g. migrant workers, truck drivers), which complicates a simplistic picture of geographic risk, nor does it account for young women being simultaneously exposed to different types of risk (e.g. AGYW who are in SDCs or who are also FSWs). Many factors that were excluded are difficult to quantify due to the scarcity of available data. However, these factors could influence oral PrEP duration (among seasonal workers, for example) and adherence as well as programme monitoring and cost. Also, most of the countries in our analysis plan to offer oral PrEP to additional high‐risk populations, including MSM, PWID, and seasonal, fisher and other vulnerable workers. We were unable to include these populations in our analysis due to the lack of reliable data to characterize them across all countries included in this analysis; however, this by no means implies that these are not important populations for PrEP provision. Additional data should be collected on these populations so that they can be included in future modelling studies.

The cost‐effectiveness results should be viewed as illustrative and interpreted with extreme caution since unit cost estimates were not derived from country‐specific primary costing exercises and did not reflect any differences in implementation models that might influence costs between the countries and across populations served. The PrEP unit costs used in this study (ranging from US$106–US$236) are somewhat higher than those previously reported (USD$87–$144) 33, 34, 35, 36, due to differences in implementation models studied and cost categories included in the different costing studies. In addition, we included optimistic assumptions for the effectiveness of and adherence to PrEP. Lower efficacy and adherence would lead to lower cost‐effectiveness and impact than estimated in this modelling exercise. Our analysis studied the impact and cost‐effectiveness from 2017 to 2030. Although useful for proximal decision making, this period is insufficient to adequately observe the full benefit of oral PrEP, as PrEP is being scaled up during the same time period that impact is being observed.

## 5 Conclusions

Although the results of our analysis can position oral PrEP as a strategic option for combination prevention programmes, they cannot fully inform more nuanced decision making, such as the selection of one risk group or intervention over another, without a better understanding of the country context (e.g. risk assessment and service delivery structures). The results of our analysis highlight challenges facing countries rolling out oral PrEP—particularly, the trade‐offs between overall impact and cost‐effectiveness that will need to be considered as they choose which populations to prioritize. As clinical and operational evidence pertaining to PrEP continues to accumulate, we expect to see revisions to WHO recommendations and rollout guidelines as well as country‐level policies and implementation plans.

Overall, our results show that oral PrEP rollout has the potential to substantially impact HIV epidemics, particularly in certain settings—averting between 3 and 8% of HIV infections in these countries between 2018 and 2030 if the UNAIDS 90‐90‐90 targets are achieved. Our results underscore the importance of tailoring rollout to specific contexts and using prioritization to maximize potential impact and cost‐effectiveness. They also emphasize the need to improve data availability and risk assessment tools to help countries make informed population prioritization decisions.

As countries decide to begin rolling out PrEP in the context of combination prevention, they must consider how implementation will affect the health system capacity, supply chain management and service delivery, particularly as they relate to ambitious ART scale‐up targets. This model and the results of our analysis can help countries evaluate the impact and cost‐effectiveness of different scale‐up scenarios.

## Competing interests

The authors declare no competing interests.

## Authors' contributions

CP, MS and KK wrote the manuscript. CP, MS, RG, MH, MR, SM, EN, DC and KK designed the analysis. CP, MS, RG and KK conducted the analysis. CP, MS, JD, RG, MH, MR, SM, EN, DC and KK reviewed and approved the manuscript.

## Abbreviations

AGYW, adolescent girls and young women; AIDS, acquired immune deficiency syndrome; AIM, AIDS Impact Model; ART, antiretroviral therapy; ARV, antiretroviral drug; FSW, female sex worker; HIV, human immunodeficiency virus; HP+, Health Policy Plus project; IPM, Incidence Patterns Model; MC, male circumcision; MSM, men who have sex with men; PEPFAR, U.S. President's Plan for Emergency AIDS Relief; PrEP, pre‐exposure prophylaxis; PWID, people who inject drugs; SDC, serodiscordant couple; SOAR, Supporting Operational AIDS Research project; USAID, U.S. Agency for International Development; VMMC, voluntary medical male circumcision; WHO, World Health Organization.

## Supporting information


**File S1.** Additional Model Details

**File S2.** Goals Modelled HIV Incidence, General Population, 2017

**File S3.** Cost Analysis and Unit Cost Data

**File S4.** S‐shaped Scale‐up Pattern Assumed for PrEP Coverage for Each Indicated Population in the Modelling Exercise

**File S5.** Number of HIV infections averted and cost per infection averted due to oral PrEP scale‐up (Scenario 4, rollout to FSWs, SDCs, and medium‐risk AGYW in all regions) from 2018 to 2030, by treatment scenario.
