# Supplementary material for: Modelling impact and cost‐effectiveness of oral pre‐exposure prophylaxis in 13 low‐resource countries
Source: J Int AIDS Soc. 2020 Feb 28;23(2):e25451. doi: 10.1002/jia2.25451 (PMC7048876; doi:10.1002/jia2.25451)
Supplement: Supplementary file 1 — File S1. Additional Model Details [file JIA2-23-e25451-s001.docx]

# Supporting Information File S1: Additional Model Details

This Word document contains supporting information for the article “Modelling impact and cost-effectiveness of oral pre-exposure prophylaxis in 13 low-resource countries.” Specifically, the document provides additional detail on two modelling tools—the Goals Model and the Incidence Patterns Model (IPM)—used in constructing our integrated modelling approach. It also includes the information used to adapt Goals, the IPM correction factors, and the stepwise linear regression method used to identify explanatory variables.

### 1.1 Goals

The Goals model is a dynamic, compartmental model that simulates HIV transmission and related morbidity and mortality for the adult population (ages 15 to 49 years). This population is structured into five mutually exclusive risk categories: low-risk heterosexual (men and women reporting a single partner in the last year), medium-risk heterosexual (men and women with more than one partner in the last year), high-risk heterosexual (female sex workers [FSWs] and their male clients), men who have sex with men (MSM), and people who inject drugs (PWID). Goals is part of the Spectrum suite of models [1] directly linked to the AIDS Impact Model (AIM) within Spectrum and uses AIM’s HIV progression structure—capturing disease progression through CD4 categories, which are the basis of antiretroviral therapy (ART) eligibility, initiation, and coverage levels, and mortality patterns. Goals has been used to study the cost and impact of national strategic plans and global strategies. Sources for Goals inputs are outlined in Table S1.1 and the 2017 ART and male circumcision coverage rates used in the Goals model are shown in Table S1.2. The final values of the Goals model parameters adjusted during model calibration are shown in Table S1.3.

Table S1.1 Data Used to Fit Goals Model

| Data Source | Data used |
| --- | --- |
| Validated country AIM files obtained from UNAIDS (http://www.unaids.org/en/dataanalysis/datatools/spectrum-epp) | HIV prevalence trends (general population) |
| Demographic and Health Surveys (DHS); AIDS Indicator Surveys (AIS) | Sexual behavior for general population (percent not sexually active, age at first sex, condom use, percent married, and sexually transmitted infection prevalence) |
|  | Population size and condom use for higher-risk heterosexual population (with a non-marital, non-cohabiting partner) |
|  | Population size and condom use for men who paid for sexual intercourse |
| UNAIDS data book (http://www.unaids.org/sites/default/files/media_asset/unaids-data-2018_en.pdf) | Population size, HIV prevalence, condom use, and intervention coverage for MSM, FSW, and PWID populations |

Table S1.2. 2017 Coverage Levels for ART and Male Circumcision

|  | ART coverage, women aged 15 and above | ART coverage, men aged 15 and above | Male circumcision coverage |
| --- | --- | --- | --- |
| Lesotho | 59.8% | 43.4% | 72.3% |
| Eswatini | 87.9% | 80.6% | 19.1% |
| Mozambique | 69.3% | 49.8% | 64.5% |
| Zambia | 77.7% | 68.0% | 43.9% |
| Zimbabwe | 67.1% | 51.3% | 14.3% |
| Malawi | 74.2% | 58.0% | 27.8% |
| Namibia | 81.9% | 60.6% | 30.8% |
| Uganda | 77.9% | 66.2% | 40.0% |
| Kenya | 70.8% | 53.0% | 92.6% |
| Nigeria | 35.0% | 20.5% | 98.9% |
| Tanzania | 72.5% | 48.4% | 80.3% |
| Haiti | 59.9% | 49.3% | 5.8% |
| Ethiopia | 63.6% | 63.0% | 91.3% |

Source: ART coverage is extracted from the Spectrum/AIM files based on country-validated service delivery data inputs and the modeled HIV-positive population in AIM. Male circumcision coverage in Spectrum/Goals was extracted from StatCompiler.com, reflecting the most recent DHS or AIS.

Table S1.3. Final Values of Parameters Adjusted in Model Calibration, 2017

|  | Low-risk | | | Medium-risk | | | Female Sex Workers | | |
| --- | --- | --- | --- | --- | --- | --- | --- | --- | --- |
|  | Population Size* | HIV Incidence | Condom Use | Population Size* | HIV Incidence | Condom Use | Population Size* | HIV incidence | Condom Use |
| Lesotho | 50.4% | 3.2% | 17.1% | 35.0% | 2.2% | 76.3% | 1.1% | 12.3% | 89.9% |
| Eswatini | 34.8% | 1.8% | 30.0% | 43.9% | 1.8% | 70.0% | 1.0% | 4.2% | 87.4% |
| Mozambique | 52.3% | 0.4% | 3.0% | 35.7% | 1.6% | 28.1% | 4.1% | 6.3% | 76.0% |
| Zambia | 73.4% | 0.8% | 4.1% | 12.8% | 1.5% | 29.4% | 0.5% | 9.9% | 78.5% |
| Zimbabwe | 52.0% | 0.7% | 3.9% | 28.2% | 0.6% | 79.0% | 1.4% | 3.7% | 89.8% |
| Malawi | 72.5% | 0.5% | 1.9% | 13.2% | 0.6% | 61.9% | 0.7% | 1.2% | 85.0% |
| Namibia | 41.7% | 0.3% | 10.6% | 40.1% | 0.5% | 65.0% | 1.7% | 3.4% | 75.0% |
| Uganda | 69.1% | 0.2% | 3.2% | 16.6% | 0.7% | 33.7% | 1.4% | 4.0% | 69.4% |
| Kenya | 45.4% | 0.2% | 3.1% | 38.8% | 0.2% | 66.9% | 1.4% | 3.8% | 91.9% |
| Nigeria | 62.2% | 0.2% | 2.1% | 22.6% | 0.2% | 48.6% | 0.7% | 3.4% | 98.1% |
| Tanzania | 63.2% | 0.1% | 2.3% | 21.8% | 0.2% | 56.4% | 1.3% | 2.6% | 78.0% |
| Haiti | 23.9% | 0.1% | 5.1% | 53.2% | 0.1% | 58.9% | 4.4% | 0.9% | 89.0% |
| Ethiopia | 72.3% | 0.0% | 1.0% | 3.7% | 0.3% | 37.7% | 0.8% | 2.2% | 81.0% |

*As a proportion of the entire female population aged 15 to 49. The size of the population “not at risk” is not shown. When this population is included, the population sizes for each country add up to 100%.

### 1.2 Incidence Patterns Model adjustment factors

IPM is a transmission model that uses a Bayesian statistical model to synthesize prior assumptions and actual data on HIV prevalence, risk, and incidence in key population groups in a single year. We used IPM to disaggregate the population by factors known to be associated with HIV acquisition, including sex, marital status, geographic location, male circumcision status, ART status, and key risk behaviors [1]. This information was used to generate adjustment factors to the impact of oral pre-exposure prophylaxis (PrEP) within Goals to allow incorporation of additional risk groups and analyses based on subnational geography. Figure S1.1 illustrates how IPM adjustment factors were applied to disaggregate the existing Goals risk groups to generate the populations studied in this analysis.

Adjustments were made to account for SDCs and AGYW at elevated risk, who form high-incidence subgroups of stable couple and medium-risk groups, respectively, in Goals. These adjustments are based on relative population size and relative incidence, which come directly from IPM.

Figure S1.1. Disaggregation of Goals Risk Groups


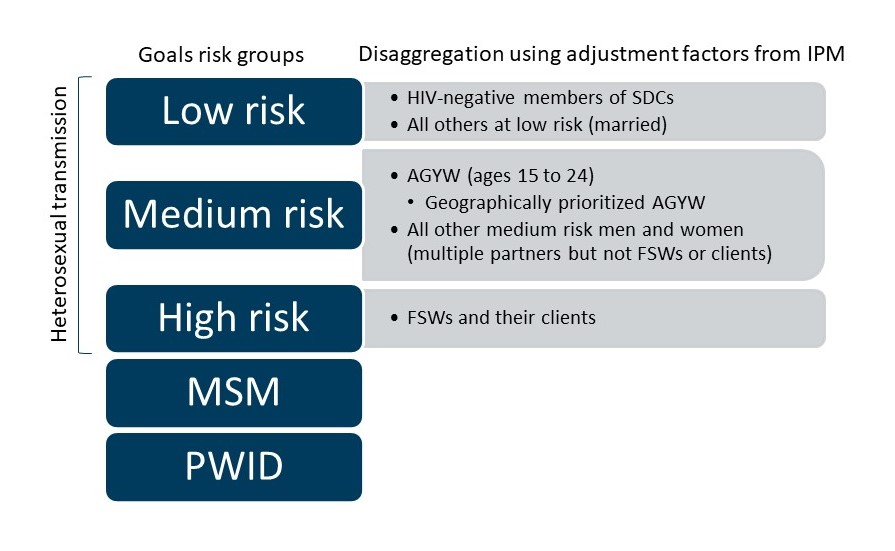


Table S1.4 shows the inputs used to generate IPM adjustment factors. Table S1.5 illustrates the IPM adjustment factors used in our modelling approach. These adjustment factors are applied as follows:

*(1+K*R_p_*R_i_)/(1+R_p_*R_i_)*

where

*K* is the reduction in incidence due to PrEP, which is equal to PrEP efficacy (0.90 in this analysis) multiplied by adherence (0.90 in this analysis) multiplied by PrEP coverage (varies over time).

*R_p_* is the relative population size of the population to be provided with PrEP

*R_i_* is the relative incidence or the population to be provided with PrEP

This entire factor is multiplied by the Goals-modelled incidence to model the impact of PrEP.

IPM uses results from trials or observational studies to develop initial estimates of incidence by population group and then applies a Bayesian framework to estimate the distribution of new infections that best matches these data and official estimates of the total number of new adult infections. For our analysis, information on population size and characteristics was drawn from the most recent Demographic and Health Survey (DHS) with HIV results available on the date of the analysis [2]. Population sizes and HIV prevalence estimates for FSWs, MSM, and PWID were obtained from recent key populations studies in respective countries (see Table S1.4). For Nigeria, for which HIV data were not collected in its DHS, the incidence and population size adjustment factors were derived by averaging adjustment factors from Cameroon and Ghana IPM models. Assumptions underpinning the transmission mechanism in the IPM model are not linked to analogous assumptions made in Goals. Both models produce compatible overall incidence estimates in the base year, which we have taken to be 2017.

Annual oral PrEP impact outcomes and costs for SDCs were also adjusted downward to account for the likely duration of six months before which the HIV-positive partner (to be initiated on ART immediately upon diagnosis) is expected to achieve viral suppression, after which additional protection offered by oral PrEP will be minimal.

Table S1.4. Inputs Used to Generate Incidence Patterns Model Adjustment Factors

| **Country** | **Model** | **Data 1 (HIV prevalence and new infections in population aged 15 to 49 years** | **Data 2 (Proportions of key populations and HIV prevalence among them)** | **Data 3: HIV prevalence distribution by age, marital status, and male circumcision** | **Data 3: HIV prevalence distribution by age, marital status, and male circumcision** |
| --- | --- | --- | --- | --- | --- |
| Lesotho | IPM, Ref #1 | Spectrum File (UNAIDS estimates 2017) | Ref#3 | Lesotho, DHS 2014 | Ref #1, #3 |
| Eswatini |  |  | Ref #4 | Swaziland DHS, 2006 | Ref #1, #4 |
| Mozambique |  |  | Ref #5 | Mozambique AIS, 2015 | Ref #1, #5 |
| Zambia |  |  | Ref #6 | Zambia DHS, 2013–2014 | Ref #1, #6 |
| Zimbabwe |  |  | Ref #7 | Zimbabwe DHS, 2015 | Ref #1, #7 |
| Malawi |  |  | Ref #1 | Malawi DHS, 2015–2016 | Ref #1 |
| Namibia |  |  | Ref #8 | Namibia DHS, 2013 | Ref #1, #8 |
| Uganda |  |  | Ref #9 | Uganda DHS, 2011 | Ref #1, #9 |
| Kenya |  |  | Ref #10 | Kenya DHS 2008–2009 | Ref #1, #10 |
| Nigeria |  |  | Ref #11, #12 | Cameroon DHS, 2011 and Ghana DHS, 2014 | Ref #1, #11, #12 |
| Tanzania |  |  | Ref #13 | Tanzania DHS, 2012–2013 | Ref #1, #13 |
| Haiti |  |  | Ref #14 | Haiti DHS, 2012 | Ref #1, #14 |
| Ethiopia |  |  | Ref #15 | Ethiopia DHS, 2016 | Ref #1, #15 |

Table S1.5. Incidence Patterns Model Adjustment Factors

|  | **SDCs within the Low-Risk Group** | | **AGYW within the Medium-Risk Group** | | **Geographic Prioritization of AGYW** | |
| --- | --- | --- | --- | --- | --- | --- |
| **Country** | **R_p_** | **R_i_** | **R_p_** | **R_i_** | **R_p_** | **R_i_** |
| Lesotho | 8.0% | 373.8% | 62.0% | 65.9% | 20.12% | 66.03% |
| Eswatini | 7.7% | 1332.8% | 84.4% | 358.5% | 21.31% | 266.07% |
| Mozambique | 4.4% | 1604.5% | 91.4% | 553.4% | 19.87% | 371.01% |
| Zambia | 6.6% | 568.6% | 66.3% | 231.9% | 10.98% | 198.19% |
| Zimbabwe | 5.9% | 562.0% | 49.3% | 112.6% | 10.85% | 154.91% |
| Malawi | 10.9% | 271.0% | 73.2% | 155.1% | 12.23% | 216.06% |
| Namibia | 5.9% | 380.0% | 37.0% | 68.4% | 5.85% | 101.11% |
| Uganda | 4.4% | 1604.5% | 91.4% | 553.4% | 19.87% | 371.01% |
| Kenya | 2.2% | 2126.1% | 53.6% | 668.7% | 14.08% | 544.18% |
| Nigeria | 2.4% | 1001.0% | 50.6% | 138.3% | 8.54% | 226.58% |
| Tanzania | 5.5% | 849.3% | 67.8% | 495.5% | 10.58% | 447.68% |
| Haiti | 1.7% | 823.8% | 60.6% | 127.4% | 5.92% | 243.18% |
| Ethiopia | 0.7% | 2215.7% | 64.9% | 165.7% | 8.55% | 474.13% |

SDCs, serodiscordant couples; AGYW, adolescent girls and young women; R_p_, population adjustment factor; R_i_, incidence adjustment factor.

## Sources

1. Bórquez A, Cori A, Pufall EL, Kasule J, Slaymaker E, Price A, et al. The Incidence Patterns Model to estimate the distribution of new HIV infections in sub-Saharan Africa: development and validation of a mathematical model. PLoS Med. 2016;13(9):e1002121.

2. DHS Program website [Internet]. Rockville, MD: The DHS Program: Demographic and Health Surveys; [cited 2017 Oct 21]. Available from: <http://www.dhsprogram.com>.

3. U.S. President’s Emergency Plan for AIDS Relief (PEPFAR). Lesotho country operational plan (COP) 2016: strategic direction summary. Washington, DC: PEPFAR; 2016.

4. U.S. Agency for International Development | Project SEARCH (Supporting Evaluation and Research to Combat HIV/AIDS), Task Order 2: Research to Prevention (R2P) project. HIV among female sex workers and men who have sex with men in Swaziland: a combined report of quantitative and qualitative studies. Baltimore, MD: Johns Hopkins Bloomberg School of Public Health Center for Communication Programs; 2013.

5. Nalá R, Cummings B, Horth R, Inguane C, Benedetti M, Chissano M, et al. Men who have sex with men in Mozambique: identifying a hidden population at high-risk for HIV. AIDS Behav. 2015;19(2):393–404.

6. Avert. Global information and education on HIV and AIDS [Internet]. Brighton, UK: Avert; c1986-2017 [cited 2017 Oct 27]. HIV and AIDS in Zambia [about 3 screens]. Available from: <https://www.avert.org/professionals/hiv-around-world/sub-saharan-africa/zambia>

7. U.S. President’s Emergency Plan for AIDS Relief (PEPFAR). Zimbabwe country operational plan, COP 2017: strategic direction summary. Washington, DC: PEPFAR; 2017.

8. U.S. President’s Emergency Plan for AIDS Relief (PEPFAR). Namibia country operational plan, COP 2017: strategic direction summary. Washington, DC: PEPFAR; 2017.

9. Uganda AIDS Commission, The Uganda HIV/AIDS Partnership. Uganda HIV and AIDS country progress report, July 2015–June 2016. Kampala, Uganda: The Republic of Uganda; 2015.

10. U.S. President’s Emergency Plan for AIDS Relief (PEPFAR). Kenya country operational plan (COP) 2017: strategic direction summary. Washington, DC: PEPFAR; 2017.

11. U.S. Agency for International Development | Project SEARCH, Task Order 2: R2P project. Examining risk factors for HIV and access to services among female sex workers and men who have sex with men in Cameroon. Baltimore, MD: Johns Hopkins Bloomberg School of Public Health Center for Communication Programs; 2014.

12. U.S. President’s Emergency Plan for AIDS Relief (PEPFAR). Ghana country operational plan (COP) 2016: strategic direction summary. Washington, DC: PEPFAR; 2016.

13. Ministry of Health and Social Welfare, National AIDS Control Programme. HIV and STI biological and behavioral survey, 2013: a study of female sex workers in seven regions: Dar es Salaam, Iringa, Mbeya, Mwanza, Shinyanga, Tabora, and Mara. Dar es Salaam: United Republic of Tanzania; 2013.

14. Ministère de la Santé Publique et de la Population. Haiti VIH 2014: surveillance biologique et comportementale utilisant la méthodologie « respondent driven sampling » (RDS) auprès des hommes ayant des relations sexuelles avec d’autres hommes (HARSAH). Port au Prince, Haiti: Organisation Haïtienne de Marketing Social pour la Santé; 2015.

15. U.S. President’s Emergency Plan for AIDS Relief (PEPFAR). Ethiopia country/regional operational plan (COP/ROP) 2017: strategic direction summary. Washington, DC: PEPFAR; 2017.
