# Supplementary material for: Modelling impact and cost‐effectiveness of oral pre‐exposure prophylaxis in 13 low‐resource countries
Source: J Int AIDS Soc. 2020 Feb 28;23(2):e25451. doi: 10.1002/jia2.25451 (PMC7048876; doi:10.1002/jia2.25451)
Supplement: Supplementary file 2 — File S2. Goals Modelled HIV Incidence, General Population, 2017 [file JIA2-23-e25451-s002.docx]

# Supporting Information File S2: Goals Modelled HIV Incidence, General Population (2017)

This Word document contains supporting information for the article “Modelling impact and cost-effectiveness of oral pre-exposure prophylaxis in 13 low-resource countries.” Specifically, the document contains a table that provides Goals modelled 2017 HIV incidence for the general population (aged 15 to 49 years).

Table S2. Modelled HIV Incidence, General Population, 2017

|  | **Women**  **(aged 15 to 49)** | **Men**  **(aged 15 to 49)** |
| --- | --- | --- |
| **Lesotho** | 2.3% | 1.3% |
| **Eswatini** | 1.6% | 0.9% |
| **Mozambique** | 0.9% | 0.6% |
| **Zambia** | 0.8% | 0.5% |
| **Zimbabwe** | 0.6% | 0.4% |
| **Malawi** | 0.5% | 0.3% |
| **Namibia** | 0.4% | 0.3% |
| **Uganda** | 0.3% | 0.2% |
| **Kenya** | 0.2% | 0.1% |
| **Nigeria** | 0.2% | 0.1% |
| **Tanzania** | 0.2% | 0.1% |
| **Haiti** | 0.1% | 0.1% |
| **Ethiopia** | 0.1% | 0.0% |
