# Supplementary material for: Modelling impact and cost‐effectiveness of oral pre‐exposure prophylaxis in 13 low‐resource countries
Source: J Int AIDS Soc. 2020 Feb 28;23(2):e25451. doi: 10.1002/jia2.25451 (PMC7048876; doi:10.1002/jia2.25451)
Supplement: Supplementary file 3 — File S3. Cost Analysis and Unit Cost Data [file JIA2-23-e25451-s003.docx]

# Supporting Information File S3: Cost Analysis and Unit Cost Data

This Word document contains details on the derivation of the country-specific oral PrEP unit costs used in the article “Modelling impact and cost-effectiveness of oral pre-exposure prophylaxis in 13 low-resource countries.”

Costs of antiretroviral drugs for each of the 13 countries included in our analysis—Eswatini, Ethiopia, Haiti, Kenya, Lesotho, Malawi, Mozambique, Namibia, Nigeria, Tanzania, Uganda, Zambia, and Zimbabwe—were obtained from the Global Price Reporting Mechanism [1] on November 28, 2017. Costs for adherence, demand generation, and personnel were scaled to reflect differences in labour costs based on the ratio of the 2017 gross national income per capita for Kenya to that of the other country. The gross national income per capita is a measure of each country’s average labour costs and was downloaded for each country from the World Bank’s gross national income per capita data catalogue [2] on November 28, 2017. The costs for training, labs, equipment, other commodities, and overhead costs were left unchanged from those collected in the Kenya study.

## Sources

1. Global Price Reporting Mechanism [Internet]. Geneva: World Health Organization; c2011 [cited 2017 Nov 28]. Available from: <http://apps.who.int/hiv/amds/price/hdd/>

2. International Comparison Program database (GNI per capita) [Internet]. Washington, DC: The World Bank. CC-BY 4.0 [cited 2017 Nov 28]. Available from: <https://data.worldbank.org/indicator/NY.GNP.PCAP.PP.CD>
