# Supplementary material for: Modelling impact and cost‐effectiveness of oral pre‐exposure prophylaxis in 13 low‐resource countries
Source: J Int AIDS Soc. 2020 Feb 28;23(2):e25451. doi: 10.1002/jia2.25451 (PMC7048876; doi:10.1002/jia2.25451)

# Supporting Information File S4: S-shaped Scale-up Pattern Assumed for PrEP Coverage for Each Population Assumed to be Provided with PrEP in the Modelling Exercise

This Word document contains supporting information for the article “Modelling impact and cost-effectiveness of oral pre-exposure prophylaxis in 13 low-resource countries.” Specifically, the document contains a figure that illustrates the scale-up of PrEP coverage over time assumed in this modelling exercise.

Figure S4. Percent of Each Priority Population Covered by PrEP


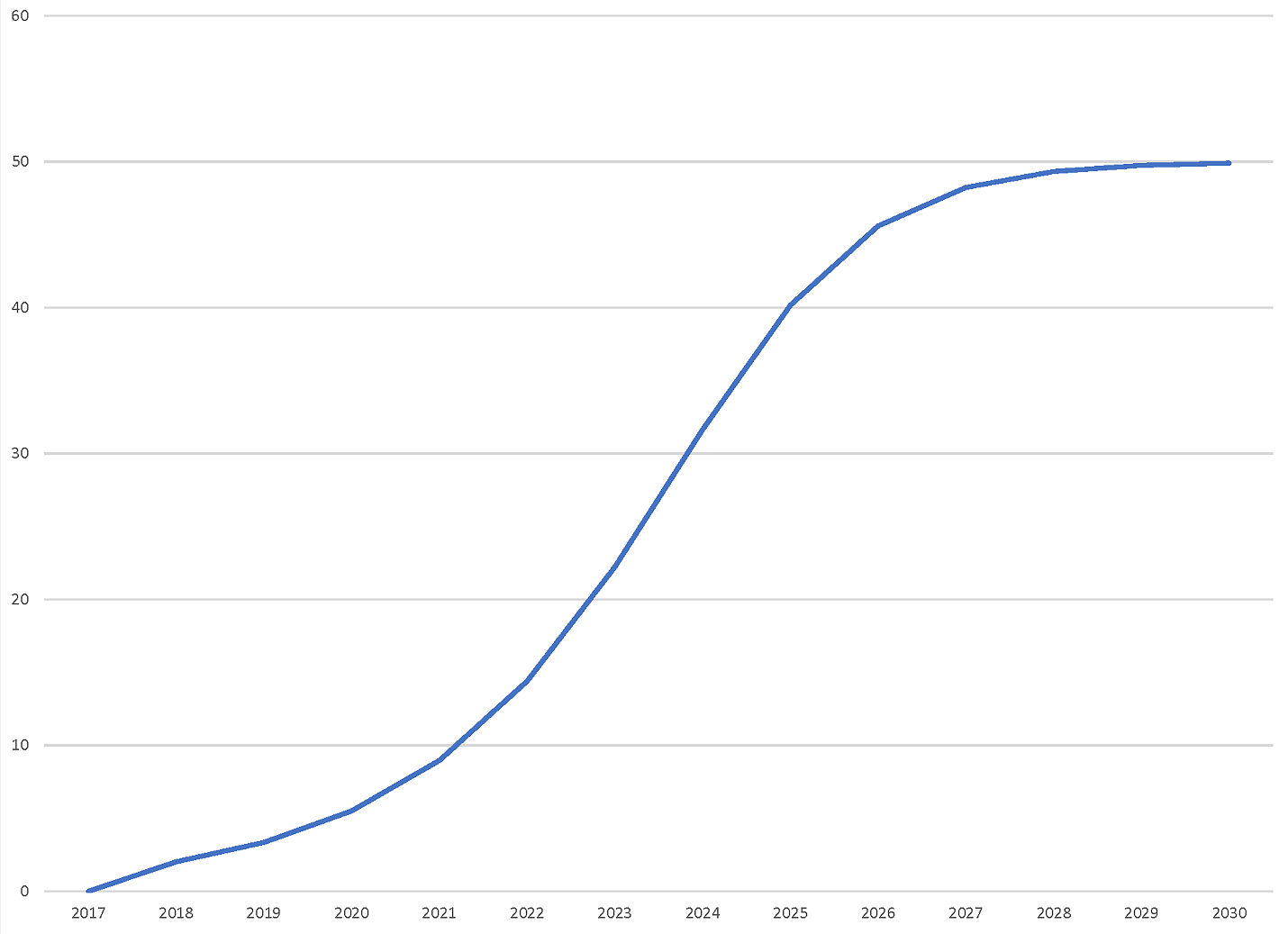

Supplement: Supplementary file 4 — File S4. S‐shaped Scale‐up Pattern Assumed for PrEP Coverage for Each Indicated Population in the Modelling Exercise [file JIA2-23-e25451-s004.docx]
