# Supplementary material for: Modelling impact and cost‐effectiveness of oral pre‐exposure prophylaxis in 13 low‐resource countries
Source: J Int AIDS Soc. 2020 Feb 28;23(2):e25451. doi: 10.1002/jia2.25451 (PMC7048876; doi:10.1002/jia2.25451)
Supplement: Supplementary file 5 — File S5. Number of HIV infections averted and cost per infection averted due to oral PrEP scale‐up (Scenario 4, rollout to FSWs, SDCs, and medium‐risk AGYW in all regions) from 2018 to 2030, by treatment scenario. [file JIA2-23-e25451-s005.docx]

# Supporting Information File S5: Number of HIV infections averted and cost per infection averted due to oral PrEP scale-up (Scenario 4, rollout to FSWs, SDCs, and medium-risk AGYW in all regions) from 2018 to 2030, by treatment scenario.

This Word document contains supporting information for the article “Modelling impact and cost-effectiveness of oral pre-exposure prophylaxis in 13 low-resource countries.” Specifically, the document contains a table that provides supplemental detail on analyses included in the article.

Table S5. HIV infections Averted and Cost per HIV Infection Averted by Scenario 4, 2018–2030, By Treatment Scenario

|  | Number of HIV infections averted | | | Cost per HIV infection averted (USD) | | |
| --- | --- | --- | --- | --- | --- | --- |
| Country | 90-90-90 by 2020 | 90-90-90 by 2030 | Continue current coverage | 90-90-90 by 2020 | 90-90-90 by 2030 | Continue current coverage |
| Eswatini | 4,000 | 7,000 | $9,000 | $12,000 | $5,000 | $4,000 |
| Ethiopia | 17,000 | 20,000 | $36,000 | $21,000 | $17,000 | $9,000 |
| Haiti | 2,000 | 3,000 | $7,000 | $192,000 | $131,000 | $50,000 |
| Kenya | 24,000 | 31,000 | $64,000 | $75,000 | $57,000 | $28,000 |
| Lesotho | 5,000 | 7,000 | $16,000 | $12,000 | $7,000 | $3,000 |
| Malawi | 17,000 | 22,000 | $46,000 | $17,000 | $13,000 | $6,000 |
| Mozambique | 37,000 | 97,000 | 145,000 | $16,000 | $6,000 | $4,000 |
| Namibia | 2,000 | 2,000 | 4,000 | $54,000 | $44,000 | $19,000 |
| Nigeria | 23,000 | 48,000 | 134,000 | $176,000 | $82,000 | $30,000 |
| Tanzania | 20,000 | 27,000 | 57,000 | $61,000 | $44,000 | $21,000 |
| Uganda | 36,000 | 43,000 | 84,000 | $17,000 | $14,000 | $7,000 |
| Zambia | 30,000 | 37,000 | 77,000 | $10,000 | $8,000 | $3,000 |
| Zimbabwe | 14,000 | 25,000 | 49,000 | $19,000 | $10,000 | $5,000 |
| Total | 231,000 | 369,000 | 728,000 | $682,000 | $438,000 | $189,000 |
